# Analysing the Temperature Effect on the Competitiveness of the Amine Addition versus the Amidation Reaction in the Epoxidized Oil/Amine System by MCR-ALS of FTIR Data

**DOI:** 10.1155/2011/401216

**Published:** 2011-06-26

**Authors:** Vanessa del Río, M. Pilar Callao, M. Soledad Larrechi

**Affiliations:** Chemometrics, Qualimetrics, and Nanosensors Group, Analytical Chemistry and Organic Chemistry Department, Rovira i Virgili University, Marcel*·*lí Domingo s/n, 43007 Tarragona, Spain

## Abstract

The evaluation of the temperature effect on the competitiveness between the amine addition and the amidation reaction in a model cure acid-catalysed reaction between the epoxidized methyl oleate (EMO), obtained from high oleic sunflower oil, and aniline is reported. The study was carried out analysing the kinetic profiles of the chemical species involved in the system, which were obtained applying multivariate curve resolution-alternating least squares (MCR-ALS) to the Fourier transform infrared spectra data obtained from the reaction monitoring at two different temperatures (60°C and 30°C). At both experimental temperatures, two mechanisms were postulated: non-autocatalytic and autocatalytic. The different behaviour was discussed considering not only the influence of the temperature on the amidation reaction kinetic, but also the presence of the homopolymerization of the EMO reagent.

## 1. Introduction

The development of environmentally compatible polymers is one of the current challenges in polymer chemistry. In this sense, epoxy resins from vegetable oils are extremely promising as environmentally friendly polymers for industrial applications because they share many of the characteristics of conventional petro-chemical based epoxy resins [1].

It is generally admitted that, when epoxide monomers are cured with amines, the addition of the amine is the strongly predominating reaction proceeding in two steps. 


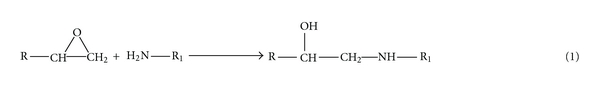
 
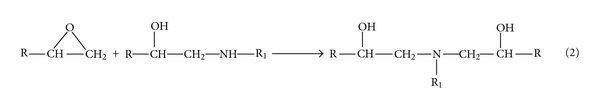


According to the type of the epoxide-amine system and to the experimental conditions, etherification reaction (3) can be done [2]. With an excess of epoxide, at high temperature and in the presence of Lewis bases, inorganic bases, or Lewis acids catalysts, homopolymerization of epoxides (4) also takes place [2]. These two parallel reactions promote the presence of ether groups. 

If the epoxide monomers (or prepolymers) contain an ester functional group, as occurs in the epoxidized oils, it is possible to consider that an amide is also formed (5) as a consequence of the reaction between the amine and the ester group. 

The existence of the homopolymerization and etherification reactions is related to such characteristics of the experimental conditions, as the temperature, the concentration of the reagents, and the presence of catalysts. However, the amidation reaction is only related to the ester-containing monomer used. 

As a consequence of the dependence of the morphology and the properties of the final product on the curing process, there has been worldwide research interest in elucidating the reaction mechanism and in quantifying the kinetics of epoxy resin cure reactions using several techniques as differential scanning calorimetry (DSC) [3], thermogravimetric analysis (TGA) [4], fluorescence [5], Raman spectroscopy [6], nuclear magnetic resonance (RMN) [7], high-resolution liquid chromatography (HPLC) [8], near infrared spectroscopy (NIR) [9], and Fourier transform infrared spectroscopy (FTIR) [10]. 

Over the past decade, FTIR has evolved rapidly and has become one of the techniques of choice to monitor *in situ* a reaction [11–13]. Usually, the information obtained from an FTIR spectrum is analysed univariately, relating the changes in a characteristic band to the global advance reaction degree. However, this kind of analysis does not provide information about the number of steps involved in the process or about the concentration of each of the chemical species and their corresponding spectra. One of the possibilities in order to obtain more information about the studied system is to use the curve resolution methods [14], as multivariate curve resolution-alternating least squares (MCR-ALS) [15–19], which allow a multivariate analysis of the spectra data to be carried out. 


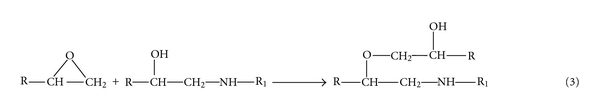
 
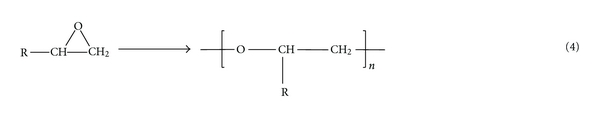
 
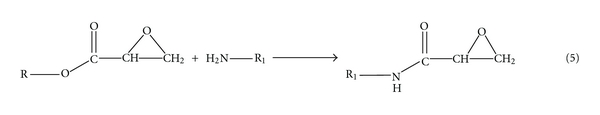


Combining FTIR-ATR spectroscopy with MCR-ALS, this report aims at evaluating the temperature effect on the mechanism of the reaction between the epoxidized methyl oleate (EMO) and aniline using borontrifluoride etherate (BF_3_·OEt_2_) as catalyst. In this system, two competitive reactions are involved: the amine addition to the oxirane ring of the epoxidized methyl oleate and the amidation reaction between the ester group of the oil and the amine group. The presence of this last kind of reaction results in cured products of poor quality [20, 21]. Thus, knowledge of the temperature effect on the kinetics of the reactions involved is required to evaluate the optimal experimental conditions of the system. 

The experimentally considered molar ratio EMO : aniline (2 : 1) reproduces the usual conditions employed in the curing processes. To analyze the temperature effect, the experiment was carried out at 60°C and at 30°C. These values were selected considering that, in a previous work [22] in which the temperature was 95°C, conversion values higher than 50% were observed from the beginning of the reaction, which does not allow the accurate analysis of the system. In addition, the homopolymerization of the EMO reagent at the two experimental temperatures was also monitored in order to evaluate the extent of this reaction. 

The resolved concentration profiles obtained by MCR-ALS were employed to identify the nature of the reaction mechanism involved at the two experimental temperatures by analysing the reaction rate versus the epoxy conversion. 

The studied reaction can be considered as a model of the curing reactions using epoxy derivatives obtained from vegetable oils. These reactions are relatively new systems and, to the best of our knowledge, no study of these characteristics is referenced in the literature. Therefore, the present study of epoxy resins contributes to better evaluation of the possibilities that vegetable oil offers as precursors of prepolymers.

## 2. Experimental

### 2.1. Materials

The epoxidized methyl oleate (EMO) was synthesized and provided by the Polymer Research Group of our department [23]. It contains 2.7 epoxide groups per molecules (determined by ^1^HNMR), and its purity was 97.89%, which was verified by elemental data analysis and ^1^H and ^13^C NMR spectra. Aniline reagent (Aldrich) and borontrifluoride etherate (BF_3_·OEt_2_) (Aldrich) were used as received.

### 2.2. Reaction Conditions and Procedure

The reaction was performed at two different temperatures: 60°C and 30°C. The reaction samples were prepared by directly mixing the necessary amounts of EMO and aniline and 2% of catalyst (BF_3_·OEt_2_) at room temperature to obtain the desired molar ratio of 2 : 1. The mixture was placed immediately on a small diamond crystal in the spectrophotometer ATR cell (FTIR 680 Plus JASCO), which was equipped with a 3000 Series High Stability Temperature Controller and an RS232 Control to take the measurements, and it was continuously purged with N_2_ during the FTIR analysis. The FTIR spectra of the reacting mixture were obtained *in situ* at equal time intervals during the isothermal cure.

In order to analyse the extent of the homopolymerization of the EMO reagent, the FTIR spectrum of a mixture of the pure EMO reactant with 2% of catalyst was monitored in the same conditions. In order to evaluate the noise, the FTIR spectra of the pure aniline and EMO reactants were also recorded during 8 hours.

### 2.3. Data Acquisition and Data Pretreatment

The data correspond to the FTIR spectra recorded throughout the reaction every 0.964 cm^−1^, between 4000 and 600 cm^−1^ in a FTIR 680 Plus JASCO spectrophotometer.

For each experiment, data at intervals of 5 minutes were acquired until the end of the reaction. The reaction was considered to be completed until no changes over time were observed in the spectra. In this way, the spectra were recorded for 250 min for the experiment at 60°C and for 480 min for the experiment at 30°C. Therefore, 50 and 96 spectra were recorded, respectively. Also, 50 and 96 FTIR spectra were recorded during the homopolymerization of the EMO reagent, and 96 FTIR spectra were acquired for the pure reactant aniline. 

All spectra were exported and converted into MATLAB binary files to carry out their mathematical treatment [24]. 

The experimental data of each experiment were arranged in matrices whose rows were the recorded spectra and whose columns were the absorbance values at different wavenumbers. Thus, the matrices obtained were **M_1_** (50 × 3528) for the experiment at 60°C, **M_2_** (96 × 3528) for the experiment at 30°C, **M_3_** (50 × 3528) and **M_4_** (96 × 3528) for the homopolymerization reaction at 60°C, and 30°C, respectively, and **M_5_** (96 × 3528) for the pure reactant aniline.

### 2.4. Soft-Modeling Multivariate Curve Resolution Methods

Soft-modeling MCR methods [25] are model-free procedures that perform the bilinear decomposition of the data matrix **D** into the product of two submatrices, **C** and **S** ^**T**^.

(1) $$\text{D}=\text{C}{\text{S}}^{\text{T}}+\text{E},$$

where **D** is the original data matrix, **C** and **S** ^**T**^ are the matrices containing the concentration and response profiles, respectively, and **E** is the matrix of residuals not explained by the model, and ideally it should be close to the experimental error. The dimensions of the four matrices are **D**(*m* × *n*), **C**(*m* × *c*), **S** ^**T**^(*c* × *n*), and **E**(*m* × *n*), where *m* and *n* are the number of rows and the number of columns of the original data matrix and *c* is the number of chemical components in the mixture or process. 

In the soft-modeling method MCR-ALS [26], **C** and **S** ^**T**^ matrices are calculated under constraints [27] solving (1) iteratively by alternating least squares (ALS) optimization, until the optimization criterion of minimizing the residuals (**D**-**CS** ^**T**^) is achieved. Before this optimization step, it is necessary to determine how many principal components or sources of variation are present in the system and to create an initial estimation of either the concentration profiles or the spectra profiles. 

In this work, ALS was applied to the matrices **D_1_** and** D_2_**, and the resolution was improved by normalizing the spectra profiles and applying the following constraints: (1) nonnegativity on the concentration profiles and (2) nonnegativity on the spectra profiles.

## 3. Results and Discussion

First, FTIR absorption spectra were analyzed and their characteristic absorption bands were assigned. Then, the concentration and spectral profiles of the chemical species involved in the reactions were solved, and the conversion of the epoxide reagent was evaluated. Finally, the quantitative analysis as well as the conversion of the epoxide in the homopolymerization of the EMO reagent was discussed and the mechanism of the studied system was investigated. 

**Figure 1:**
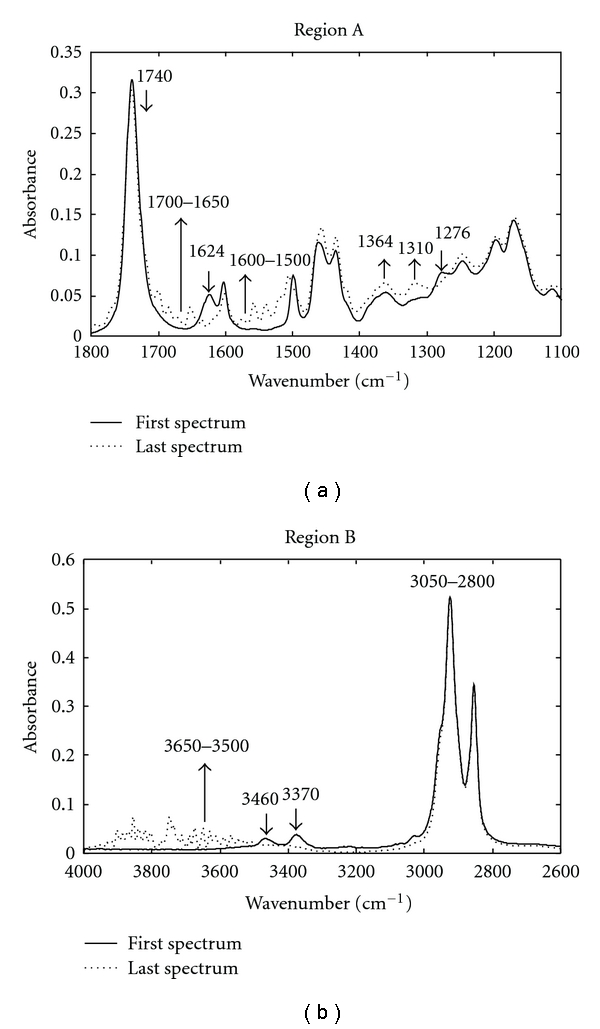
First and last FTIR-ATR of EMO/aniline system 2 : 1 recorded at *T* ^a^ = 60°C. (a) Region between 1800–1100 cm^−1^ (A) and (b) region between 4000–2600 cm^−1^ (B).

For better exposition of the results, the first and the last spectra recorded throughout the reaction between EMO and aniline at 60°C are displayed in the graphs (Figures 1(a) and 1(b)). The two wavenumber regions of particular interest in the FTIR spectra are 1800–1100 cm^−1^ and 4000–2600 cm^−1^. In order, these will be referred to as regions A and B and are shown in Figures 1(a) and 1(b), respectively. The characteristic absorption bands of the functional groups that participate in the EMO/aniline system are summarized in Table 1 [28].

In region A, Figure 1(a), it is possible to observe that, throughout the reaction time, the intensity of the characteristic absorption bands of the N–H bending vibrations at 1624 cm^−1^ characteristic of the aniline reagent, and of the C–O vibrations of the oxirane ring at 1276 cm^−1^ characteristic of the EMO reagent, decreases. On the other hand, an increase in the intensities of the absorption bands associated with some of the functional groups present in the products generated by the reactions described in equations (1)–(5) can be observed: between 1600–1500 cm^−1^ the absorption bands of the N–H bending vibrations of the secondary amine, between 1700–1650 cm^−1^ the absorption bands of the C=O stretching vibrations of the amide, and at 1364 cm^−1^ and 1310 cm^−1^ the absorption bands of the C–N vibrations of the tertiary amine. 

In region B, Figure 1(b), as the reaction progresses, it is possible to observe a decrease in the intensity of the two bands at 3460 and 3370 cm^−1^, which are associated with the N–H stretching vibrations of the aniline reagent, and an increase in the intensity of the bands between 3650 cm^−1^ and 3500 cm^−1^, which are related to the O–H stretching vibrations of the hydroxyl group present in all the products generated. 

Similar spectral changes were observed when the analysis of the spectra recorded at 30°C was carried out. However, it should be said that, at this experimental temperature, the intensities of the absorption bands between 1700 cm^−1^ ad 1650 cm^−1^ associated with the amide compound were significantly lower.

The spectroscopic analysis reflects that, at the two experimental temperatures, the main amine additions to the oxirane ring ((1) and (2)) and the amidation reaction (5) have taken place. 

**Scheme 1:**
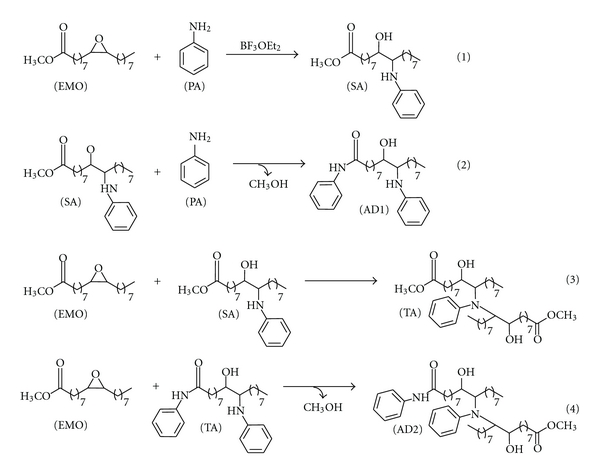
Reaction between EMO and aniline using BF_3_·OEt_2_ as catalyst (1) primary amine addition to the oxirane ring; (2) amidation reaction; (3) secondary amine addition to the oxirane ring; (4) amidation reaction. (EMO) epoxidized derivative oleic oil; (PA) aniline; (SA) secondary amine; (AD1) amide; (TA) tertiary amine; (AD2) tertiary amine.

Before carrying out the quantitative resolution of the concentration and spectral profiles of the chemical species involved in the reactions, the number of independent contributions to the variation present in the individual matrices **M_1_** and **M_2_** was evaluated by singular value decomposition (SVD) [29] (Table 2). A visual analysis of the values obtained shows that from the fifth singular value there is a large breakoff, so five singular values can be considered to be significantly different from the background noise, which according to Amhrein et al. [30] means that four independent reactions can be present. Considering the results obtained in the previous work [22], where the sequential order of the primary amine addition to the oxirane ring and of the amidation reaction was established by two-dimensional correlation spectroscopy and chemometric techniques, it can be postulated that, in the experimental conditions, these four reactions correspond to those indicated in Scheme 1. Formally, in these reactions, seven chemical species are involved. However, taking into account the experimental temperatures and that the system was monitored without interrupting the N_2_ purge of the FTIR-ATR spectrometer, it can be assumed that the methanol formed in the amidation reaction is displaced from the cell. Therefore, the number of chemical species that can be expected throughout the reaction is six. In any case, regarding the significant values (Table 2), it is evidenced that rank deficiency exists in both matrices. 

To overcome this problem and to obtain the evolution of the concentration profiles along the reaction time of the six chemical species, the multivariate curve resolution-alternating least squares method was applied to the column-wise augmented data matrices **D_1_** and **D_2_**, which were constructed with the data corresponding to the **M_1_** and **M_2_** experimental data matrices, respectively, and the pure spectra of the EMO and aniline reagents. In these augmented matrices, the number of significant singular values was six, as is shown in Table 2. The variance associated with the solutions found by MCR-ALS for the augmented matrices **D_1_** and **D_2_** was 99.94% and 98.50%, respectively. 

**Figure 2:**
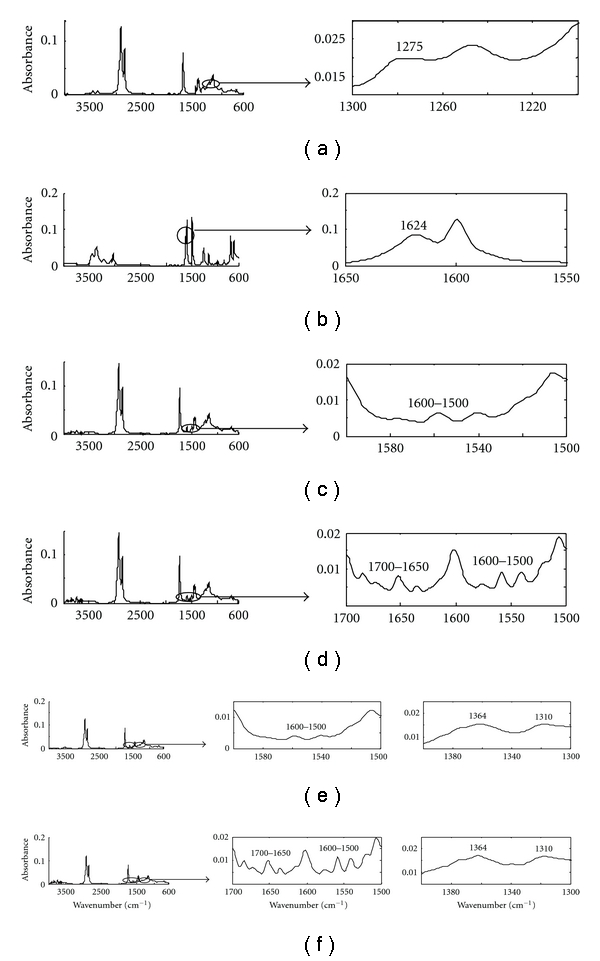
Spectral profiles of the species recovered by MCR-ALS: (a) EMO; (b) aniline; (c) secondary amine (SA); (d) amide (AD1); (e) tertiary amine (TA); (f) tertiary amine (AD2).

As example, Figure 2 shows the spectra for each of the six species recovered by MCR-ALS from the **D_1_** matrix. To obtain a better visualization, a restricted spectral zone where the characteristic spectral bands of each compound appear is amplified. The first and the second recovered spectra (A and B) contain the characteristic bands of the epoxy group at 1276 cm^−1^ and of the primary amine group at 3460, 3372 and 1624 cm^−1^, respectively. For these spectra, their similarity with the pure spectra recorded for EMO and aniline reagents was evaluated throughout the correlation coefficient, finding *r* values of 0.9910 and 0.9995, respectively. Thus, the first and the second spectra were assigned to the EMO and aniline reagents, respectively. 

The other four recovered spectra are very similar, as expected considering the chemical structure of the compounds. In all of them, there are no signals from either the primary amine or from the oxirane group in any of the representative zones, and, on the other hand, it is possible to detect the bands associated with the OH functional group (3650–3500 cm^−1^). The two recovered spectra C and D were assigned to the secondary amine (SA) and to the amide compound (AD1) formed in reactions (1) and (2) of Scheme 1, respectively. These two spectra show the characteristic spectral bands of the secondary amine functional group between 1600 and 1500 cm^−1^, and the only difference between them is observed in the wavenumber range characteristic of an amide compound between 1700 and 1650 cm^−1^, where absorbance values appear in spectra D. Finally, the last two recovered spectra (E and F) can be related to the tertiary amine compounds (TA and AD2) formed in reactions (3) and (4) of Scheme 1, respectively. These two spectra show the characteristic bands of tertiary amines, located at 1364 and 1310 cm^−1^. The difference between them falls in the spectral zones related to the secondary amine functional group (1600–1500 cm^−1^) and to the amide functional group (1700–1650 cm^−1^), where absorbance values can be only distinguished in spectrum F, as expected. 

**Figure 3:**
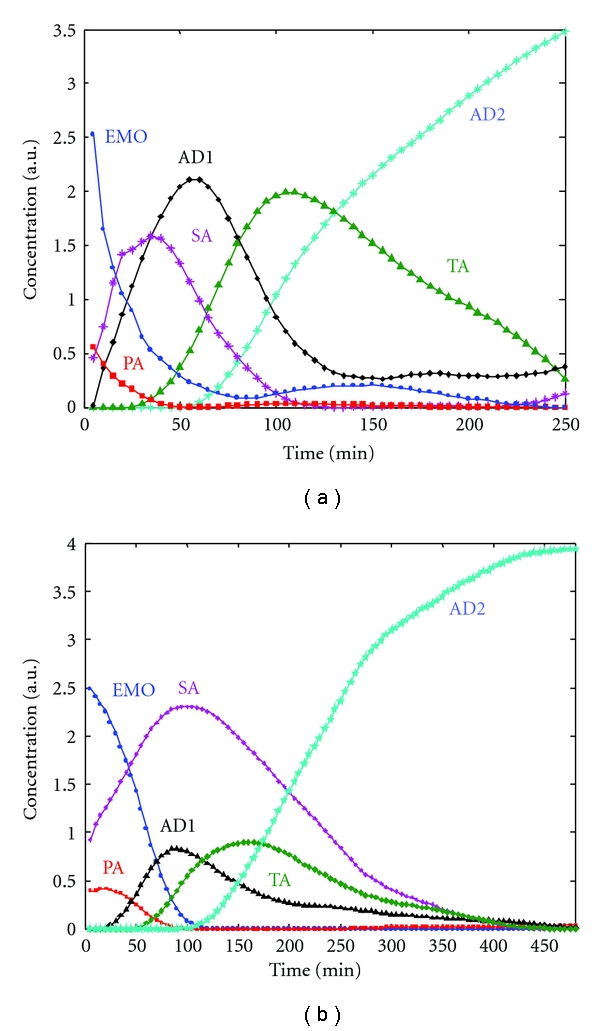
Concentration profiles of the species recovered by MCR-ALS (a) *T* ^a^ = 60°C; (b) *T* ^a^ = 30°C. (*⬤*) EMO; (■) aniline; (*) secondary amine (SA); (▲) amide (AD1); (♦) tertiary amine (TA); (*⋆*) tertiary amine (AD2).

The concentration profiles obtained by MCR-ALS for the EMO/aniline system at the two experimental temperatures (60°C and 30°C) are shown in Figures 3(a) and 3(b). It is known that the solutions obtained by MCR-ALS are not unique and around each profile exist band boundaries which contain the possible solutions that fit the experimental data equally well [31]. Therefore, the shown concentration profiles are only one of the possible solutions. However, in overall terms, these solutions seem a good reflection of the expected behavior, and they were considered useful to compare the effect of the temperature on the amine addition/amidation reactions involved in the process. 

In Figure 3, it is observed that in both experiments, from the beginning of the reaction, the concentration value of the secondary amine compound (SA) formed in the first amine addition to the oxirane ring is noteworthy. Besides, the emergence of this compound is faster at 60°C than at 30°C, achieving the maximum concentration value at 25 and at 100 minutes, respectively. 

The temperature effect on the behaviour of the amide concentration profile (AD1) generated in the amidation reaction is worthy of note. In the experiment at 60°C (Figure 3(a)), a significant change in its concentration is observed from the beginning, and it achieves a maximum value at 50 minutes. However, at this time in the experiment at 30°C (Figure 3(b)), the change in the AD1 concentration value is lower and, at no time along the reaction does it achieve the maximum observed at 60°C. In addition, comparing the evolution of these AD1 profiles with the evolution of the concentration profiles of the other chemical species in both experiments, it can be considered that at 60°C the kinetics of the amidation reaction ((2) in Scheme 1) compete with the kinetics of the primary amine addition ((1) in Scheme 1), while at 30°C the kinetics of the amidation reaction are relatively similar to the kinetics of the second amine addition ((3) in Scheme 1). 

**Figure 4:**
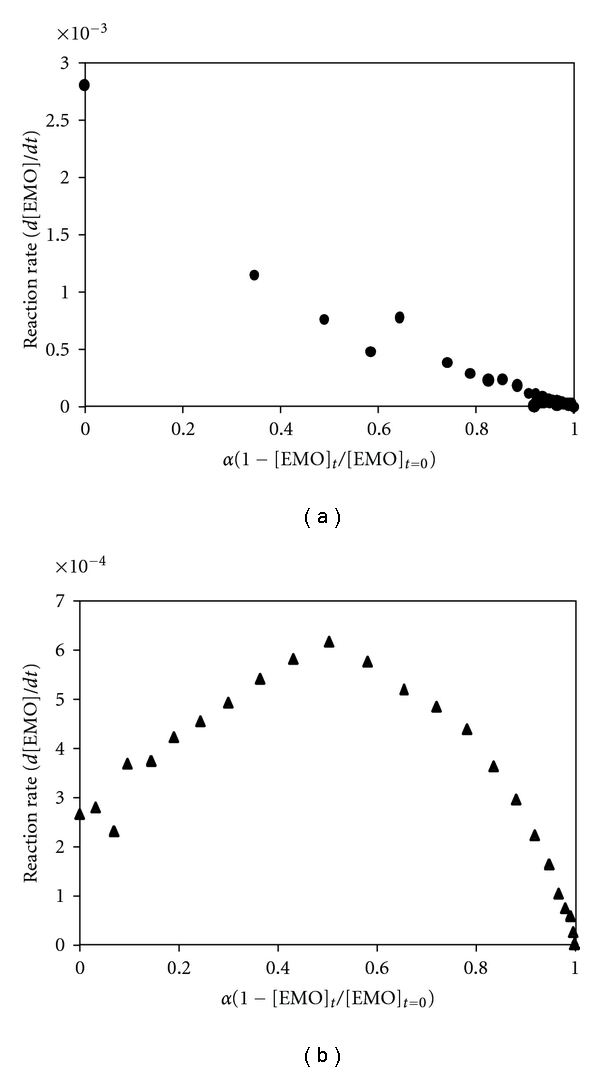
Reaction rate versus conversion (*α*): (a) 60°C; (b) 30°C.

From these results, it can be supposed that the pathway of the process is not the same at the two experimental temperatures. In order to provide a basis for understanding this consideration, the epoxy conversion (*α* = 1 − [EMO]_*t*_/[EMO]_*t*=0_) throughout the reaction time was calculated using the concentration values expressed in moles ([EMO]_*t*_) of the EMO concentration profiles, and it was plotted versus the reaction rate (*d*[EMO]/*dt*) (Figures 4(a) and 4(b)). The corresponding values in moles were obtained by dividing the EMO-recovered concentration profiles by its corresponding molecular weight. The plot corresponding to the values obtained at 30°C (Figure 4(b)) shows the characteristic behaviour of the autocatalytic reactions [32]; the reaction rate increases initially as the reaction advances, passes through a maximum, and progressively slows down tending to zero. The initial increase in the reaction rate is associated with the formation of hydroxyl groups, which acts as catalyst of the reaction. The maximum rate is observed at conversion between 40–50%, as expected for autocatalytic reactions. However, at 60°C the maximum rate appears at extremely low conversion (<0.1) and decreases with the reaction time, which led to the conclusion that in this case the mechanism is nonautocatalytic.

Two possibilities can explain the different behaviour of the system at the two experimental temperatures. Firstly, as the authors commented above, the methanol formed in the amidation reaction can be evaporated at 60°C faster than at 30°C, so the amidation reaction takes place more easily, and it is favoured at higher temperatures.

Secondly, as was indicated in the introduction, although the main reactions involved in the cure process are the amine additions to the oxirane ring, when the reaction takes place in presence of a Lewis acid as catalyst, homopolymerization reaction is also reported [2]. To evaluate this possibility, the epoxy conversion was calculated from the spectra recorded during the homopolymerization of the EMO reagent, whose data was arranged in matrices **M_3_** and **M_4_**, considering that if homopolymerization takes place, the intensity of the characteristic band of the oxirane ring at 1276 cm^−1^ diminishes, so the epoxy conversion was obtained from the disappearing of this band during the time. At the reaction times of 50 minutes and 100 minutes, where the concentration of the amide compound (AD1) was maximum for the two temperatures (Figure 3), 60°C and 30°C, respectively, the conversion values obtained were 1.88% at 60°C and 0.17% at 30°C, in agreement with the consideration that this reaction is less favoured than the amine addition reaction. However, although this reaction can be considered quantitatively neglected, in the working experimental conditions, it may play an important role in the behaviour of the amide reaction and, therefore, in the pathway of the process.

**Scheme 2:**
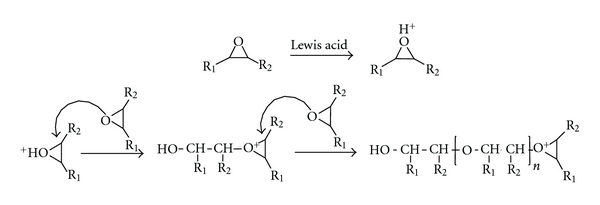
Homopolymerization of epoxide by activate chain end (ACE) mechanism. R_1_ = –(CH_2_)_7_–COOEt, R_2_ = –(CH_2_)_7_–CH_3_.

In the homopolymerization of EMO, the cationic ring opening of the epoxide could follow the activated chain-end (ACE) mechanism [33], as indicated in Scheme 2. The ACE mechanism involves nucleophilic attack of the heteroatom of the monomer to the growing chain end, which is a cationic species. As a result, polyether with pendant ester groups is formed. The presence of these ester groups could explain the high and fast increase in the concentration profile of the amide compound shown in Figure 3(a). 

These expounded likelihoods are not mutually exclusive and, in any case, from all these considerations, it is reasonable to assume that the formation of the amide compound plays an important role in the mechanism of the reaction. 

## 4. Conclusions

The importance of the temperature effect on a model curing reaction of epoxy resins from vegetable oils was successfully demonstrated by the combination of infrared spectroscopy and multivariate curve resolution-alternating least squares. The corresponding concentration and spectral profiles of the species involved in the EMO/aniline system monitored were successfully drawn, and the first ones were a valuable guide for analysing the mechanism of the process. 

Two different mechanisms depending on the temperature for the EMO/aniline system were postulated: at low temperatures, the mechanism was autocatalytic and at high temperatures was nonautocatalytic. 

It has been evidenced that higher temperatures favour the amidation reaction in the model system studied. As epoxidized vegetable oils contain ester groups when they are cured with amine hardeners, the amidation reaction will take place in some extent, what could lead to products of poor quality.

## Figures and Tables

**Table 1:** Assignment of the absorption bands in the FTIR-ATR spectra (see Figure 1).

Origin	Wavenumber (cm^−1^)	Assignment
C–O–	1276	Epoxy and oxirane ring
C–N	1310	Aromatic tertiary amine, CN stretching
C–N	1364	Aromatic tertiary amine, CN stretching
N–H	1500–1600	Secondary amine, NH bending
N–H	1624	Primary amine, NH bending
C=O	1650–1700	Amide, CO stretching
C=O	1740	Ester, CO stretching
CH_2_–	2800–3050	Methylene, CH stretching
N–H	3370	Primary amine, NH stretching
N–H	3460	Primary amine, NH stretching
O–H	3500–3650	Hydroxy group, OH stretching

**Table 2:** Rank analysis of matrices ** M** and **D**.

Number of factor	**M**_1_	**D**_1_	**M**_2_	**D**_2_
1	29.0408	29.3428	37.6861	37.9182
2	1.954	5.4823	2.9207	5.8910
3	0.5727	1.9385	2.0849	2.8395
4	0.2867	0.7053	0.3270	1.9131
5	0.2129	0.2779	0.2461	0.5935
6	0.0845	0.2263	0.0930	0.2912
7	0.0374	0.0579	0.0492	0.0706
8	0.0295	0.0339	0.0424	0.0473
